# Multiple Moderating Roles of Growth Mindset in the Links Between Teacher Autonomy Support and Learning Engagement

**DOI:** 10.3390/bs16091489

**Published:** 2026-08-26

**Authors:** Yangcun Feng, Huiling Wu, Jun Guan, Tongji Li

**Affiliations:** 1 College of Vocational and Technical Education, Tongji University, Shanghai 201804, China; 2 Shanghai Pudong Institute of Education Development, Shanghai 200127, China

**Keywords:** teacher autonomy support, learning engagement, beliefs about errors, academic relaxation, growth mindset

## Abstract

Perceived teacher autonomy support is consistently associated with student engagement, but the cognitive and emotional processes underlying this association remain insufficiently understood. Drawing on self-determination theory and control-value theory, this study examined whether beliefs about errors and academic relaxation serially mediated the association between perceived teacher autonomy support and mathematics learning engagement, and whether growth mindset moderated the paths leading to engagement. Data were collected from 1679 Chinese primary, junior secondary, senior secondary, and secondary vocational students. Confirmatory factor analysis, serial mediation analysis, and moderated serial mediation analysis were conducted. The five-factor measurement model showed acceptable fit and measurement invariance across school stages. After controlling for gender and school stage, perceived teacher autonomy support was positively associated with mathematics learning engagement. Beliefs about errors and academic relaxation showed significant specific and serial indirect associations. Growth mindset strengthened the association between perceived teacher autonomy support and mathematics learning engagement but did not robustly moderate the association between beliefs about errors and engagement. Contrary to the original hypothesis, growth mindset weakened the positive association between academic relaxation and engagement, although this association remained positive at both low and high levels of growth mindset. These findings reveal path-specific moderation and suggest that a calm, low-threat learning state may be particularly relevant to engagement among students with lower growth mindset. Because the data were cross-sectional and self-reported, causal interpretations are not warranted.

## 1. Introduction

Learning engagement reflects the behavioral, emotional, and cognitive resources that students invest in learning activities (Fredricks et al., 2004; Martins et al., 2022; Wang et al., 2021). In mathematics education, sustained engagement is particularly important because students regularly encounter difficult tasks, incorrect solutions, and the need to revise strategies. Nevertheless, students differ substantially in whether they continue to participate, think deeply, and persist after experiencing errors or difficulty. Identifying classroom conditions and psychological processes associated with mathematics learning engagement is therefore an important issue in educational research. Self-determination theory (SDT) suggests that teachers’ autonomy-supportive behaviors constitute an important classroom resource for student motivation and engagement. Autonomy-supportive teaching refers to teaching behaviors through which teachers acknowledge students’ perspectives, provide meaningful choices, explain the value of learning activities, and minimize coercive control during instruction (Ryan & Deci, 2000, 2017). Previous research has consistently reported a positive association between teacher autonomy support and student engagement (Chen et al., 2015; Froiland et al., 2016; Jiang et al., 2023; Reeve et al., 2004; Sun, 2016; Yu et al., 2016). Nevertheless, the psychological processes through which students interpret and respond to such support require further examination. In particular, students’ beliefs about errors and their emotional experiences during mathematics learning may help explain why perceived teacher autonomy support is associated with sustained engagement.

In examining the psychological process through which teacher autonomy support is associated with learning engagement, two pathways deserve closer attention. The first is cognitive: how students understand and evaluate the learning obstacles they encounter, especially the experience of making mistakes. The second is affective: the emotional states students experience during academic activities. These two dimensions are not isolated; rather, they are internally connected and logically sequential. Specifically, when students view mistakes as normal attempts and valuable forms of exploration in learning, errors are less likely to constitute a psychological threat, and students are less likely to carry an excessive psychological burden. They can therefore maintain a relatively relaxed emotional state during academic activities. Conversely, when students equate mistakes with lack of ability or failure, each error experience may arouse anxiety and tension and may even lead to avoidance, causing learning to stagnate. Prior research has shown that students’ beliefs about errors are closely associated with their adaptive emotional, motivational, and behavioral responses in error situations, and that students’ emotional experiences after success or failure are related to how they interpret errors and failure (Tulis & Ainley, 2011; Tulis et al., 2018). Academic emotions also influence students’ cognitive resources, learning strategies, self-regulation, and learning behavior (Pekrun, 2006; Pekrun et al., 2002). Thus, cognitive appraisal can be understood as an antecedent condition of emotional response, and emotional state further shapes whether students are willing to sustain learning engagement. On this basis, this study introduces beliefs about errors and academic relaxation as serial mediators. However, even within the same context of teacher autonomy support, students may not show the same pattern of sequential associations among their cognitive interpretations of errors, emotional experiences, and learning engagement. Individual differences may cause the efficacy of this mediating chain to vary across student groups. Growth mindset, that is, the implicit belief that ability can be developed through effort, strategy adjustment, and continued learning, constitutes a key boundary condition (Blackwell et al., 2007; Burnette et al., 2013; Dweck & Leggett, 1988; Yeager & Dweck, 2012).

Based on the preceding analysis, the present study examined perceived teacher autonomy support as the contextual variable, beliefs about errors and academic relaxation as serial mediators, mathematics learning engagement as the outcome, and growth mindset as a moderator. Specifically, the study addressed three questions. First, is perceived teacher autonomy support indirectly associated with mathematics learning engagement through beliefs about errors and academic relaxation? Second, does growth mindset moderate the paths in this chain leading to mathematics learning engagement? Third, does the moderating pattern of growth mindset differ across the teacher-support, cognitive, and affective pathways? By addressing these questions, the study seeks to provide a more differentiated account of the cognitive and emotional processes associated with mathematics learning engagement.

## 2. Literature Review and Research Hypotheses

### 2.1. Perceived Teacher Autonomy Support and Mathematics Learning Engagement

Teacher autonomy support is a central application of self-determination theory (SDT) in classroom teaching. According to SDT, students’ sustained learning is not merely a product of external requirements or outcome incentives; it also depends on the satisfaction of basic psychological needs for autonomy, competence, and relatedness. When teachers provide appropriate choices, explain the value of learning tasks, accept students’ perspectives, and offer feedback in a non-controlling manner, students are more likely to develop autonomous motivation and participate actively in learning (Ryan & Deci, 2000, 2017). Thus, teacher autonomy support does not simply mean reducing teacher guidance. Rather, it supports students’ agency and helps them experience meaning, control, and competence in learning activities.

Learning engagement is an integrated state of student participation in the learning process and generally includes behavioral, emotional, and cognitive engagement. It is reflected not only in classroom participation and task completion, but also in students’ positive experiences, sustained effort, and deep thinking during learning activities (Fredricks et al., 2004). Previous studies have shown that teacher autonomy support can significantly enhance students’ classroom engagement. For example, Reeve et al. (2004) found in an intervention study that increasing teachers’ autonomy-supportive behaviors improved students’ learning participation. Using longitudinal data, Yu et al. (2016) showed that basic psychological need satisfaction and school engagement sequentially mediated the association between teacher autonomy support and adolescents’ anxiety and depression. In the context of mathematics learning, Froiland et al. (2016) found that teacher autonomy support was positively associated with students’ mathematics motivation, course taking, and mathematics achievement. Studies conducted in China have also reported significant positive associations between teacher autonomy support and learning engagement among junior secondary students, as well as online learning engagement among senior secondary students (Chen et al., 2015; Jiang et al., 2023).

Accordingly, this study argues that teacher autonomy support provides an important contextual foundation for students’ learning engagement. In particular, when teachers respect students’ thinking processes, accept imperfect attempts during exploration, and guide students to continue learning through supportive feedback, students are more likely to maintain a high level of learning engagement. Therefore, this study proposes:

**Hypothesis** **1.** *Perceived teacher autonomy support is positively associated with mathematics learning engagement*.

### 2.2. The Mediating Roles of Beliefs About Errors and Academic Relaxation

Learning is often accompanied by misunderstandings, obstacles, and strategy adjustment. How students interpret errors affects their subsequent emotional responses and learning behaviors. Positive beliefs about errors emphasize that errors can serve as useful feedback in the learning process, helping students identify misunderstandings, revise learning strategies, and remain engaged. Negative beliefs about errors, in contrast, tend to frame errors as evidence of insufficient ability or learning failure, which may easily induce avoidance and defensive responses. Tulis et al. (2018) showed that positive beliefs about errors predict students’ adaptive affective, motivational, and behavioral responses in error situations.

Teacher autonomy support may be an important classroom condition for the formation of positive beliefs about errors. Supportive teachers do not merely provide opportunities for choice; they also respect students’ thinking processes, accept their imperfect attempts, and treat errors as resources for further discussion and improvement. In such a classroom environment, students are more likely to understand errors as a normal part of learning rather than as a denial of their own ability. Therefore, teacher autonomy support may be associated with learning engagement partly through students’ judgments about the meaning of errors.

Academic emotions refer to the emotional experiences that students develop during learning activities, and they can influence learning motivation, cognitive processing, and learning behavior. Pekrun et al. (2002) noted that academic emotions are closely related to students’ motivation, learning strategies, cognitive resources, self-regulation, and academic performance. Control-value theory further argues that students’ perceived control over, and value attached to, learning activities shape their academic emotions, which in turn affect subsequent learning processes (Pekrun, 2006). Prior research has also found that students’ emotional experiences after success or failure are closely related to how they interpret errors and failure (Tulis & Ainley, 2011).

In the present study, academic relaxation refers to a calm and low-threat emotional state during mathematics learning, including feeling relatively at ease in class, while completing assignments, and when facing examinations or learning tasks. It does not refer to disengagement, indifference, boredom, or reduced effort. Rather, it reflects the absence of excessive tension and psychological defensiveness. Nevertheless, the present study tests only a linear association and cannot determine whether extremely high levels of relaxation might reduce attention or effort.

Accordingly, this study argues that beliefs about errors and academic relaxation may constitute a sequential psychological pathway linking perceived teacher autonomy support with mathematics learning engagement. Teacher autonomy support helps students understand errors as usable learning information, thereby reducing the perceived threat of errors and fostering a relatively relaxed academic emotional state. Academic relaxation does not mean slackness in learning; rather, it indicates that students can continue to try, reflect, and adjust under a lower level of psychological defensiveness. Recent research has linked teacher emotional support with student academic engagement through positive academic emotions (Shen et al., 2024). Based on this reasoning, the following hypotheses are proposed:

**Hypothesis** **2.** *Beliefs about errors mediates the association between perceived teacher autonomy support and mathematics learning engagement.*

**Hypothesis** **3a.** *Academic relaxation mediate the association between perceived teacher autonomy support and mathematics learning engagement.*

**Hypothesis** **3b.** *Beliefs about errors and academic relaxation serially mediate the association between perceived teacher autonomy support and mathematics learning engagement.*

### 2.3. The Moderating Role of Growth Mindset

Growth mindset refers to individuals’ beliefs about the malleability of ability, emphasizing that ability is not fixed but can be developed through effort, strategy adjustment, and continuous learning. Dweck and Leggett (1988) proposed that individuals’ basic beliefs about the nature of ability affect their goal orientations and further shape their cognitive, emotional, and behavioral responses when they encounter difficulties and failure. Therefore, growth mindset is not merely a general positive psychological quality; rather, it is an underlying cognitive framework that influences how students interpret learning difficulties, error experiences, and external support. Existing studies have shown that growth mindset can affect students’ learning adaptation. In a longitudinal and intervention study of adolescents’ mathematics learning, Blackwell et al. (2007) found that students who believed ability could be developed were more likely to develop positive effort beliefs, attributional patterns, and learning strategies. Burnette et al.’s (2013) meta-analysis further indicated that implicit theories of ability are systematically associated with self-regulatory processes, including goal setting, goal monitoring, and goal attainment. These findings suggest that growth mindset may influence whether students can transform external support and error feedback into positive and sustained learning engagement.

On this basis, growth mindset may strengthen the association between perceived teacher autonomy support and mathematics learning engagement. For students with higher levels of growth mindset, teachers’ provision of choice, supportive feedback, and tolerance of errors may be more readily interpreted as conditions for ability development and may therefore be more strongly associated with active participation and sustained engagement. Conversely, students with lower levels of growth mindset may make less use of perceived external support when they hold relatively fixed beliefs about ability. Therefore, the following hypothesis was proposed:

**Hypothesis** **4a.** *Growth mindset positively moderates the association between perceived teacher autonomy support and mathematics learning engagement, such that the association is stronger at higher levels of growth mindset.*

In addition, growth mindset may moderate the effects of beliefs about errors and academic relaxation emotion on learning engagement. First, beliefs about errors reflect how students understand mistakes that occur during learning. When students hold positive beliefs about errors, they are more likely to regard errors as important information for identifying problems, revising their understanding, and promoting growth, and are therefore more willing to continue engaging in learning activities. However, whether such cognitive beliefs can be further translated into stable learning engagement is also influenced by growth mindset. Students with higher levels of growth mindset believe that ability can be developed. Thus, when they develop positive beliefs about errors, they are more likely to transform error feedback into motivation for subsequent effort and to maintain learning engagement through strategy adjustment. Accordingly, the following hypothesis was proposed:

**Hypothesis** **4b.** *Growth mindset positively moderates the association between beliefs about errors and mathematics learning engagement, such that the association is stronger at higher levels of growth mindset.*

Second, academic relaxation emotion is a positive, low-arousal emotional state, indicating that students feel safe, calm, and less pressured during the learning process. In general, relaxation may reduce psychological defensiveness and the depletion of cognitive resources, thereby helping students maintain sustained and focused engagement. Students with higher levels of growth mindset may be more likely to use a relaxed emotional state when approaching challenging tasks and engaging in deep cognitive processing. In other words, growth mindset may strengthen the positive effect of academic relaxation emotion on learning engagement. Accordingly, the following hypothesis was proposed:

**Hypothesis** **4c.** *Growth mindset positively moderates the association between academic relaxation and mathematics learning engagement, such that the association is stronger at higher levels of growth mindset.*

**Figure 1:**
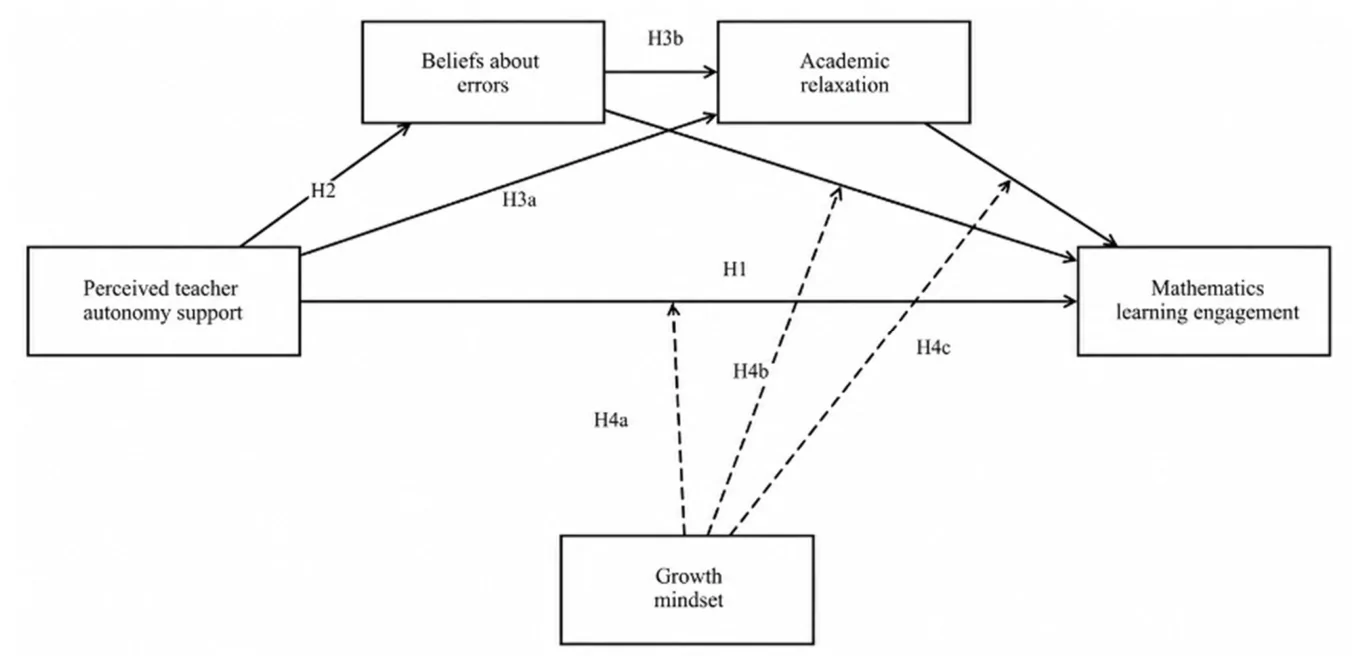
Hypothesized model. Solid arrows indicate the hypothesized direct and mediation paths, whereas dashed arrows indicate the moderating effects of growth mindset.

In sum, the hypothesized model of this study is presented in Figure 1.

## 3. Materials and Methods

### 3.1. Participants

This study was conducted across primary and secondary schools in multiple regions of China, including Shanghai, Anhui, and Shaanxi. Students were primarily recruited with the assistance of schools, which organized eligible students to participate. Prior to completing the questionnaires, participants were fully informed about the study’s theme, objectives, and significance, as well as the data privacy safeguards in place. They were explicitly notified of their right to withdraw from the study at any point, even during the completion process. Online questionnaires were distributed via the “Wenjuanxing” platform. Under the guidance of researchers or teachers, students completed these surveys independently with flexible timing, rather than through centralized in-class administration. Paper-based questionnaires were uniformly distributed by classroom teachers or researchers during breaks or self-study periods and collected on-site immediately after completion to ensure data integrity and validity.

A total of 2398 questionnaires were returned, including 2284 responses collected through Wenjuanxing and 114 paper-based responses. One questionnaire with a missing response on a focal study item and one questionnaire with an invalid gender code were excluded. Response-pattern screening was then conducted using the 21 items measuring the five focal constructs. A total of 717 questionnaires were excluded because the same response option was selected for at least 20 of the 21 focal items. The final analytic sample comprised 1679 students, yielding an analytic-sample retention rate of 70.0%. Of the retained participants, 769 were male, and 910 were female. By school stage, 692 students were in primary school, including the preparatory grade, 515 were in junior secondary school, and 472 were in senior secondary or secondary vocational school.

### 3.2. Measures

Regarding the measurement instruments, all scales except the Academic Relaxation Emotion scale were adapted from established English-language instruments. The translation and contextual adaptation procedure followed standard back-translation protocols. Initially, two doctoral candidates in Education with overseas study experience and advanced English proficiency conducted the initial translation and cultural adaptation. Subsequently, two frontline teachers reviewed the translated items to ensure linguistic accuracy and contextual relevance within the Chinese educational setting. After two rounds of revision, a pilot test was administered to 30 students in a single classroom by a frontline teacher. Data from this pilot test demonstrated satisfactory reliability and validity, justifying the launch of the large-scale formal survey. All items were rated on a five-point Likert scale ranging from 1 (strongly disagree) to 5 (strongly agree). Mean scores were calculated for each construct, with higher scores indicating higher levels of the corresponding construct.

#### 3.2.1. Perceived Teacher Autonomy Support

Perceived teacher autonomy support was assessed using three items adapted from Froiland et al. (2016) to the mathematics-learning context. The items assessed whether mathematics teachers listened to students’ ideas, accepted errors as a normal part of learning, and respected students. A sample item was, “My mathematics teacher values students’ ideas and listens carefully.” Cronbach’s α was 0.716, CFA-based ω was 0.827, composite reliability was 0.827, and the average variance extracted was 0.625.

#### 3.2.2. Beliefs About Errors

Beliefs about errors were assessed using five items adapted from Tulis et al. (2018). The items measured the extent to which students regarded mathematical errors as useful information for learning and improvement. A sample item was, “I can learn new knowledge or problem-solving strategies from mistakes in mathematics.” Higher scores indicated more adaptive beliefs about errors. Cronbach’s α was 0.933, CFA-based ω was 0.952, composite reliability was 0.952, and the average variance extracted was 0.798.

#### 3.2.3. Academic Relaxation

Academic relaxation was assessed using five items adapted from the Academic Emotions Questionnaire for Adolescents developed by Dong and Yu (2007). The items assessed students’ calmness and low-threat experiences during mathematics classes, examinations, assignments, and learning tasks. A sample item was, “I generally feel relaxed during mathematics class.” Cronbach’s α was 0.921, CFA-based ω was 0.941, composite reliability was 0.941, and the average variance extracted was 0.760.

#### 3.2.4. Mathematics Learning Engagement

Mathematics learning engagement was assessed using three items adapted from the Utrecht Work Engagement Scale–Student Form originally developed by Schaufeli et al. (2002) and subsequently validated in student samples by Gusy et al. (2019). The items reflected vigor, enthusiasm, and positive absorption in mathematics learning. A sample item was, “I feel energetic when studying mathematics or attending mathematics class.” Cronbach’s α was 0.934, CFA-based ω was 0.954, composite reliability was 0.954, and the average variance extracted was 0.873.

#### 3.2.5. Growth Mindset

Mathematics growth mindset was assessed using five items adapted from MacDonnell Mesler et al. (2021) and contextualized for mathematics learning. The measure was grounded in the implicit-theories framework developed by Dweck and colleagues. The items assessed students’ beliefs that mathematical ability could be developed through effort and learning. A sample item was, “I can improve my mathematics ability through effort.” Cronbach’s α was 0.865, CFA-based ω was 0.911, composite reliability was 0.911, and the average variance extracted was 0.674.

### 3.3. Data Analysis

Statistical analyses were conducted using IBM SPSS Statistics 26.0, R 4.6.1, and Python 3.12.13. The R packages lavaan 0.7-2, semTools 0.5-9, psych 2.6.5, and haven 2.5.5 were used for the measurement analyses and data processing. Descriptive statistics, Pearson correlations, item analyses, and internal-consistency estimates were first calculated.

Because the measurement items used five-point ordered response categories, confirmatory factor analyses were estimated using the robust weighted least-squares estimator. The hypothesized five-factor model was compared with two alternative four-factor models and a single-factor model. Composite reliability, average variance extracted, and heterotrait–monotrait ratios were used to evaluate convergent and discriminant validity. Measurement invariance across school stages was examined sequentially at the configural, threshold, and loading levels.

Hypothesis 1 was examined using regression analysis with gender and school stage entered as covariates. The serial and moderated serial mediation specifications corresponded to Hayes’ Model 6 and Model 89, respectively, and were estimated using custom Python routines with HC3 standard errors and bootstrap confidence intervals. Continuous variables were standardized before the interaction terms were constructed. Because heteroscedasticity was detected, HC3 heteroscedasticity-consistent standard errors and large-sample normal-approximation z tests were used. Indirect effects, conditional indirect effects, and indices of moderated mediation were estimated using 5000 bootstrap samples. The bootstrap procedure produced 5000 successful samples and no failed samples. Simple slopes were calculated at one standard deviation below and above the mean of growth mindset. Statistical significance was evaluated using two-sided tests with α = 0.05 and 95% confidence intervals. To evaluate possible differences associated with data screening, the retained sample was compared with the 717 straight-line-excluded respondents in terms of gender, school stage, and survey mode using Pearson chi-square tests, with Cramér’s V reported as the effect-size measure. Online completion times were compared using the Mann–Whitney U test and rank-biserial correlation. Sensitivity analyses were then conducted using four alternative screening specifications: excluding only respondents who selected the same option for all 21 focal items, excluding respondents who selected the same option for at least 36 of all 37 measurement items, and additionally excluding online completion times below 60 or 90 s. The focal regression, indirect, and interaction effects were re-estimated under each specification using 5000 bootstrap samples.

## 4. Results

### 4.1. Common Method Bias Test

Harman’s single-factor analysis was conducted as an initial diagnostic, but it was not treated as sufficient evidence for excluding common method variance. More importantly, the hypothesized five-factor CFA model fitted the data substantially better than the single-factor model, indicating that the observed covariance structure could not be adequately represented by one general factor. We additionally attempted an orthogonal unmeasured latent method-factor model in which all items loaded on their theoretical factors and on a common method factor. However, the information matrix could not be inverted, and standard errors and robust fit indices were unavailable; the solution was therefore regarded as unstable and potentially underidentified. Given that all core constructs were assessed through self-reports collected at one time point and no independent marker variable was available, common method variance cannot be fully ruled out.

### 4.2. Sample Screening and Attrition Analysis 

Comparison analyses showed that the retained and straight-line-excluded respondents differed significantly in gender, school stage, survey mode, and online completion time. The demographic differences were small in magnitude, with Cramér’s V values ranging from 0.088 to 0.132. Straight-line respondents were more likely to be male, to have completed the questionnaire online, and to have substantially shorter completion times. Among online respondents, the median completion time was 165 s for the retained sample and 81 s for the excluded sample, Mann–Whitney *U* = 884,506.5, *p* < 0.001, rank-biserial *r* = 0.578. Detailed results are reported in Table A1.

### 4.3. Measurement Model, Reliability, and Validity

The hypothesized five-factor model showed acceptable fit, χ^2^(179) = 1288.713, CFI = 0.988, TLI = 0.986, RMSEA = 0.061, 90% CI [0.058, 0.064], and SRMR = 0.028. The model fitted the data substantially better than the model combining academic relaxation and learning engagement, the model combining beliefs about errors and learning engagement, and the single-factor model. CFA-based reliability and composite reliability were acceptable for all constructs. All HTMT values were below 0.85; the highest value was 0.830 between academic relaxation and mathematics learning engagement, indicating acceptable discriminant validity, although academic relaxation and mathematics learning engagement were strongly related. Measurement invariance analyses supported configural, threshold, and loading invariance across school stages (Table 1 and Table 2).

### 4.4. Descriptive Statistics and Correlations

To preliminarily examine the relationships among the core variables, descriptive statistics and Pearson correlation analyses were conducted for perceived teacher autonomy support, beliefs about errors, academic relaxation, mathematics learning engagement, and growth mindset. The results are presented in Table 3. Overall, the directions of the correlations among the variables were consistent with theoretical expectations. Perceived teacher autonomy support was significantly and positively correlated with beliefs about errors, academic relaxation, learning engagement, and growth mindset, indicating that students who perceived higher levels of teacher autonomy support tended to report more positive beliefs about errors, greater academic relaxation, higher learning engagement, and stronger growth mindset. Beliefs about errors were significantly and positively correlated with academic relaxation, learning engagement, and growth mindset, suggesting that students who were more likely to understand errors as feedback with learning value also tended to experience lower threat and higher learning engagement. Academic relaxation was strongly and positively correlated with learning engagement, indicating that students in a less anxious and less defensive state were more likely to maintain sustained participation in learning. Growth mindset was also significantly and positively correlated with the other variables, providing a preliminary basis for testing its moderating role.

It should be noted that correlation analysis only reflects linear associations among variables and cannot be used to infer causal relationships. These results were used mainly to determine whether the directions of the relationships among variables were consistent with theoretical expectations and to provide a basis for the subsequent serial mediation and moderated mediation analyses.

### 4.5. Association Between Perceived Teacher Autonomy Support and Mathematics Learning Engagement

After controlling for gender and school stage, perceived teacher autonomy support was positively associated with mathematics learning engagement, *B* = 0.562, HC3 *SE* = 0.027, *z* = 20.816, *p* < 0.001, 95% CI [0.509, 0.615], *R*^2^ = 0.318. Hypothesis 1 was therefore supported.

### 4.6. Serial Mediation Analysis

The serial mediation model was estimated using the procedure described in Section 3.3, with the three-item measure of perceived teacher autonomy support and with gender and school stage included as covariates. HC3 standard errors and 5000 bootstrap samples were used to estimate the path coefficients and indirect associations. The results are presented in Table 4.

**Figure 2:**
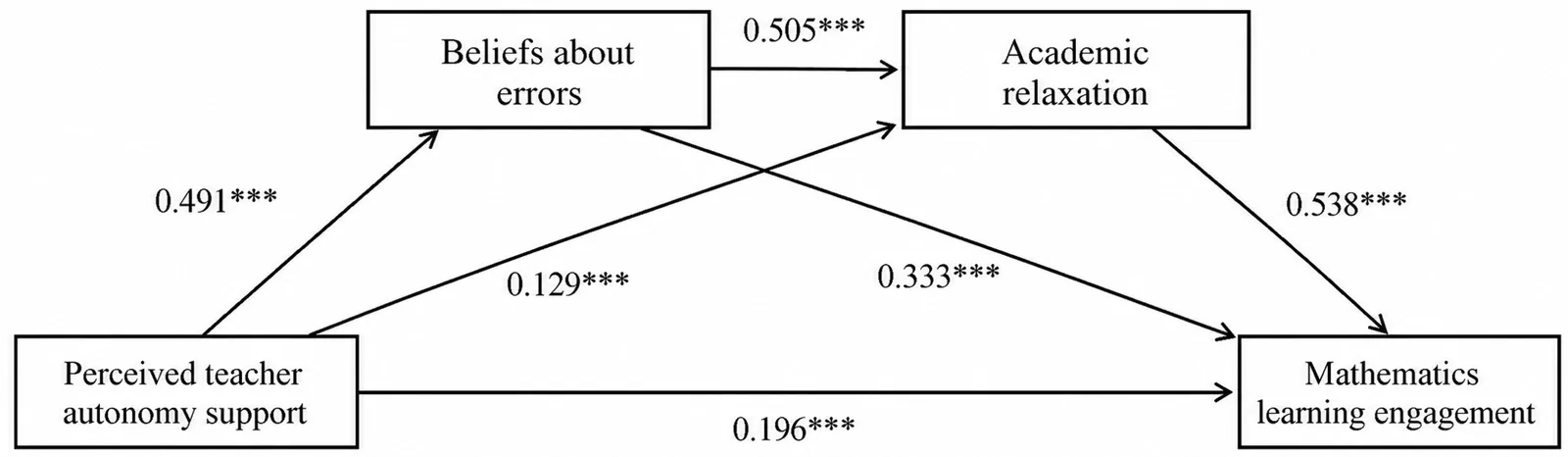
Path diagram of mediation coefficients. Note. *** *p* < 0.001.

As shown in Table 4, perceived teacher autonomy support was positively associated with beliefs about errors, *B* = 0.491, *p* < 0.001. Perceived teacher autonomy support and beliefs about errors were positively associated with academic relaxation, *Bs* = 0.129 and 0.505, respectively, *p* < 0.001. In the engagement equation, perceived teacher autonomy support, beliefs about errors, and academic relaxation were positively associated with mathematics learning engagement, *Bs* = 0.196, 0.333, and 0.538, respectively, *p* < 0.001. Gender and school stage were included as covariates (Figure 2).

The total association between perceived teacher autonomy support and mathematics learning engagement was 0.562, 95% CI [0.509, 0.615]. After beliefs about errors and academic relaxation were included, the direct association was 0.196, 95% CI [0.144, 0.247]. The total indirect association was 0.366, 95% bootstrap CI [0.316, 0.415]. The indirect association through beliefs about errors was 0.163, 95% bootstrap CI [0.125, 0.204], supporting Hypothesis 2. The indirect association through academic relaxation was 0.069, 95% bootstrap CI [0.036, 0.106], supporting Hypothesis 3a. The serial indirect association through beliefs about errors and academic relaxation was 0.134, 95% bootstrap CI [0.109, 0.159], supporting Hypothesis 3b (Table 5). 

### 4.7. Moderated Serial Mediation Analysis

The moderated serial mediation model was estimated using the procedure described in Section 3.3. All focal continuous variables were standardized before the interaction terms were constructed. Gender and school stage were included as covariates, and HC3 standard errors and 5000 bootstrap samples were used. 

As shown in Table 6, the interaction between perceived teacher autonomy support and growth mindset was significant, *B* = 0.040, HC3 *SE* = 0.018, *z* = 2.193, *p* = 0.028, 95% CI [0.004, 0.075]. The interaction between beliefs about errors and growth mindset did not reach statistical significance, *B* = 0.044, HC3 *SE* = 0.027, *z* = 1.650, *p* = 0.099, 95% CI [−0.008, 0.097]. The interaction between academic relaxation and growth mindset was significant and negative, *B* = −0.086, HC3 *SE* = 0.028, *z* = −3.099, *p* = 0.002, 95% CI [−0.140, −0.032]. Thus, Hypothesis 4a was supported, Hypothesis 4b was not supported, and Hypothesis 4c was not supported because the observed direction was opposite to the prediction (Table 7).

#### 4.7.1. Moderating Role of Growth Mindset in the Association Between Perceived Teacher Autonomy Support and Mathematics Learning Engagement

**Figure 3:**
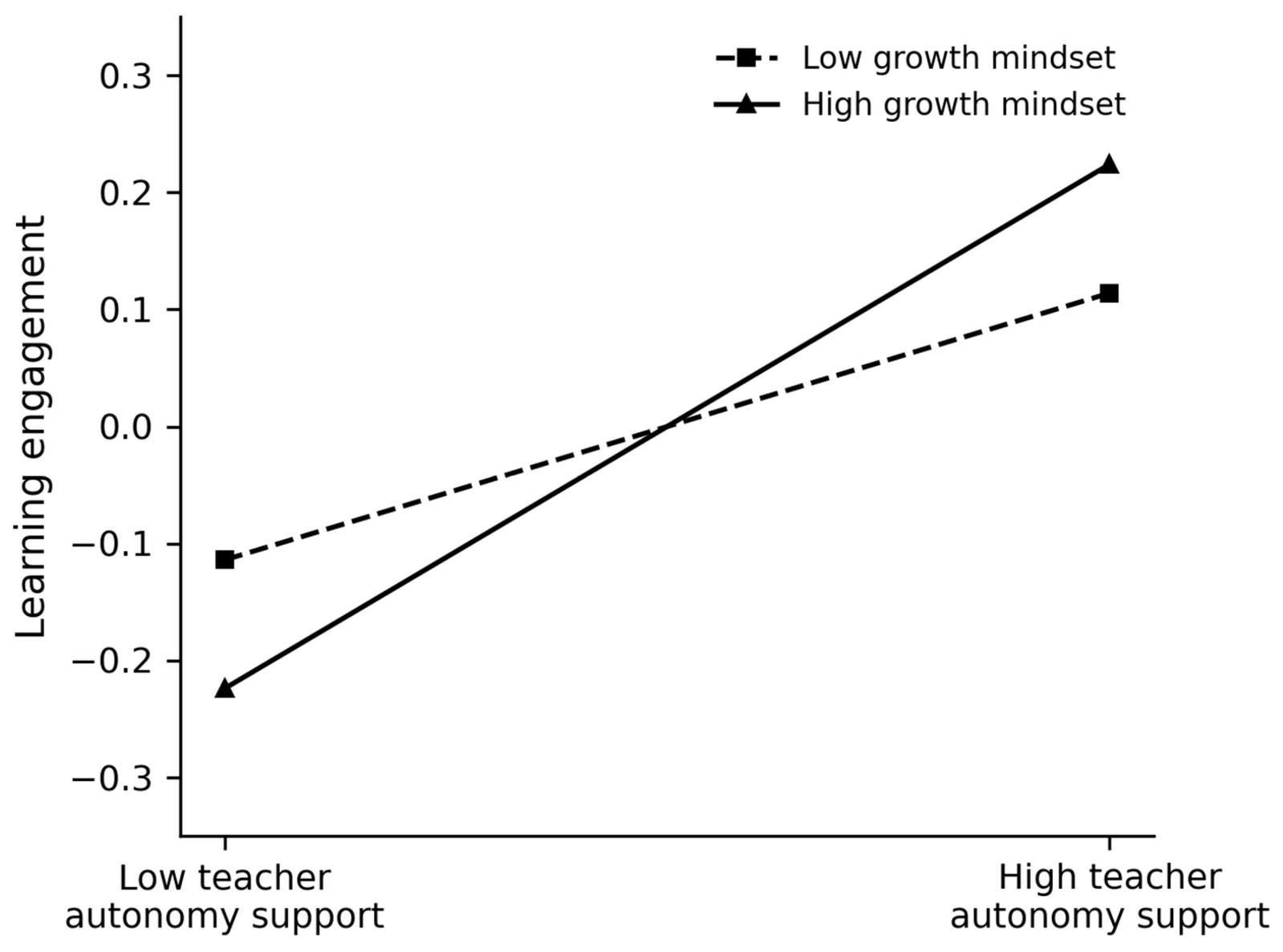
Moderating Role of Growth Mindset in the Association Between Perceived Teacher Autonomy Support and Mathematics Learning Engagement. Note. All continuous variables were standardized. Low and high growth mindset represent −1 SD and +1 SD from the mean, respectively.

Simple-slope analyses indicated that perceived teacher autonomy support was positively associated with mathematics learning engagement at both low and high levels of growth mindset. At one standard deviation below the mean of growth mindset, the association was *B* = 0.135, HC3 *SE* = 0.022, *z* = 6.039, *p* < 0.001, 95% CI [0.091, 0.178]. At one standard deviation above the mean, the association was stronger, *B* = 0.214, HC3 *SE* = 0.036, *z* = 6.016, *p* < 0.001, 95% CI [0.144, 0.284]. These results supported Hypothesis 4a (Figure 3).

#### 4.7.2. Moderating Role of Growth Mindset in the Association Between Beliefs About Errors and Mathematics Learning Engagement

**Figure 4:**
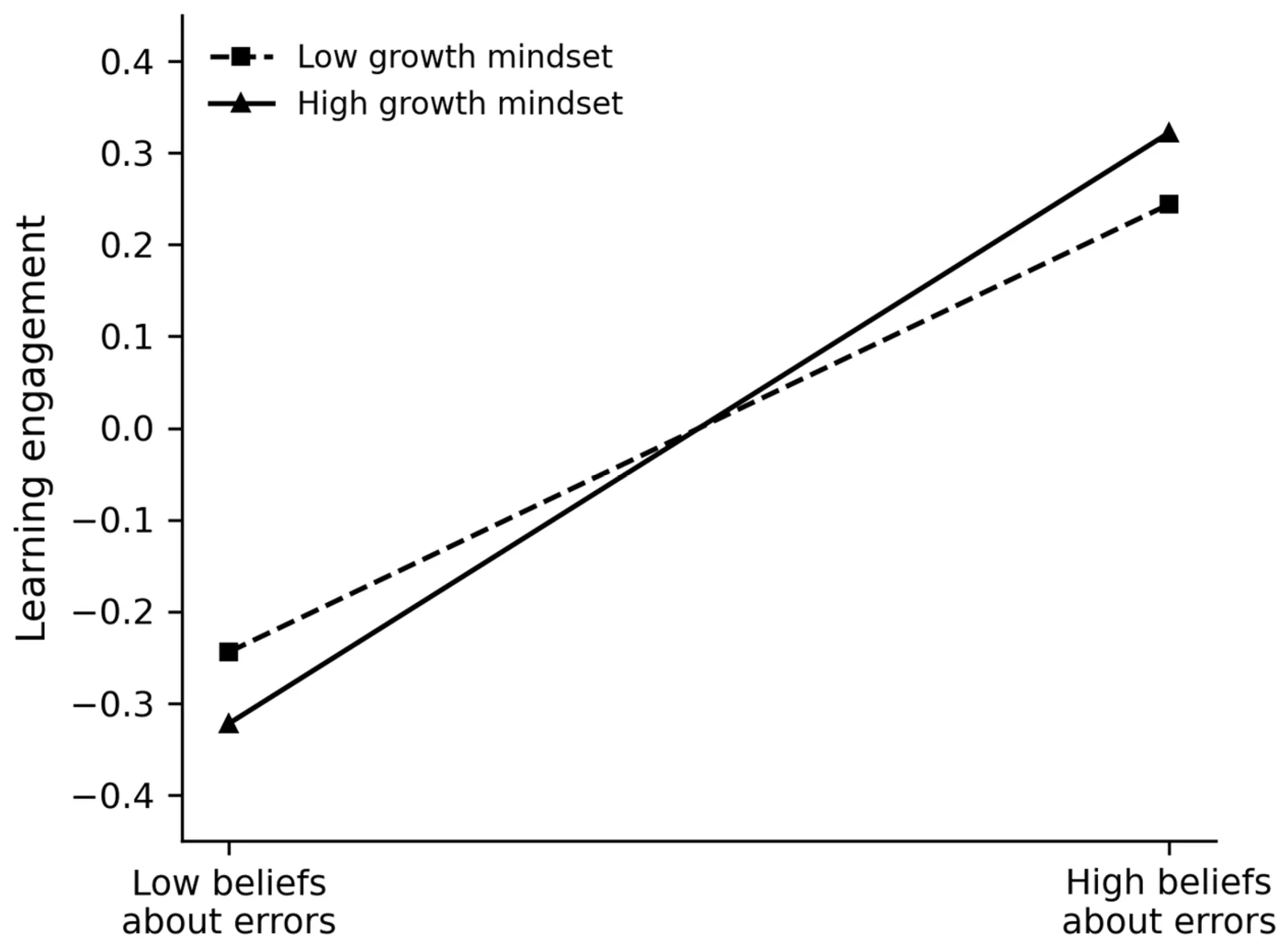
Descriptive Simple Slopes for the Association Between Beliefs About Errors and Mathematics Learning Engagement at Low and High Levels of Growth Mindset. Note. The interaction was not statistically significant in the full-sample HC3 analysis; the figure is provided for descriptive purposes only.

The interaction between beliefs about errors and growth mindset did not reach statistical significance in the full-sample HC3 analysis, *B* = 0.044, HC3 *SE* = 0.027, *z* = 1.650, *p* = 0.099, 95% CI [−0.008, 0.097]. Although beliefs about errors were positively associated with engagement at both low growth mindset, *B* = 0.236, HC3 *SE* = 0.039, 95% CI [0.159, 0.313], and high growth mindset, *B* = 0.324, HC3 *SE* = 0.040, 95% CI [0.245, 0.403], the difference between these slopes was not statistically robust. Hypothesis 4b was therefore not supported (Figure 4).

#### 4.7.3. Moderating Role of Growth Mindset in the Association Between Academic Relaxation and Mathematics Learning Engagement

**Figure 5:**
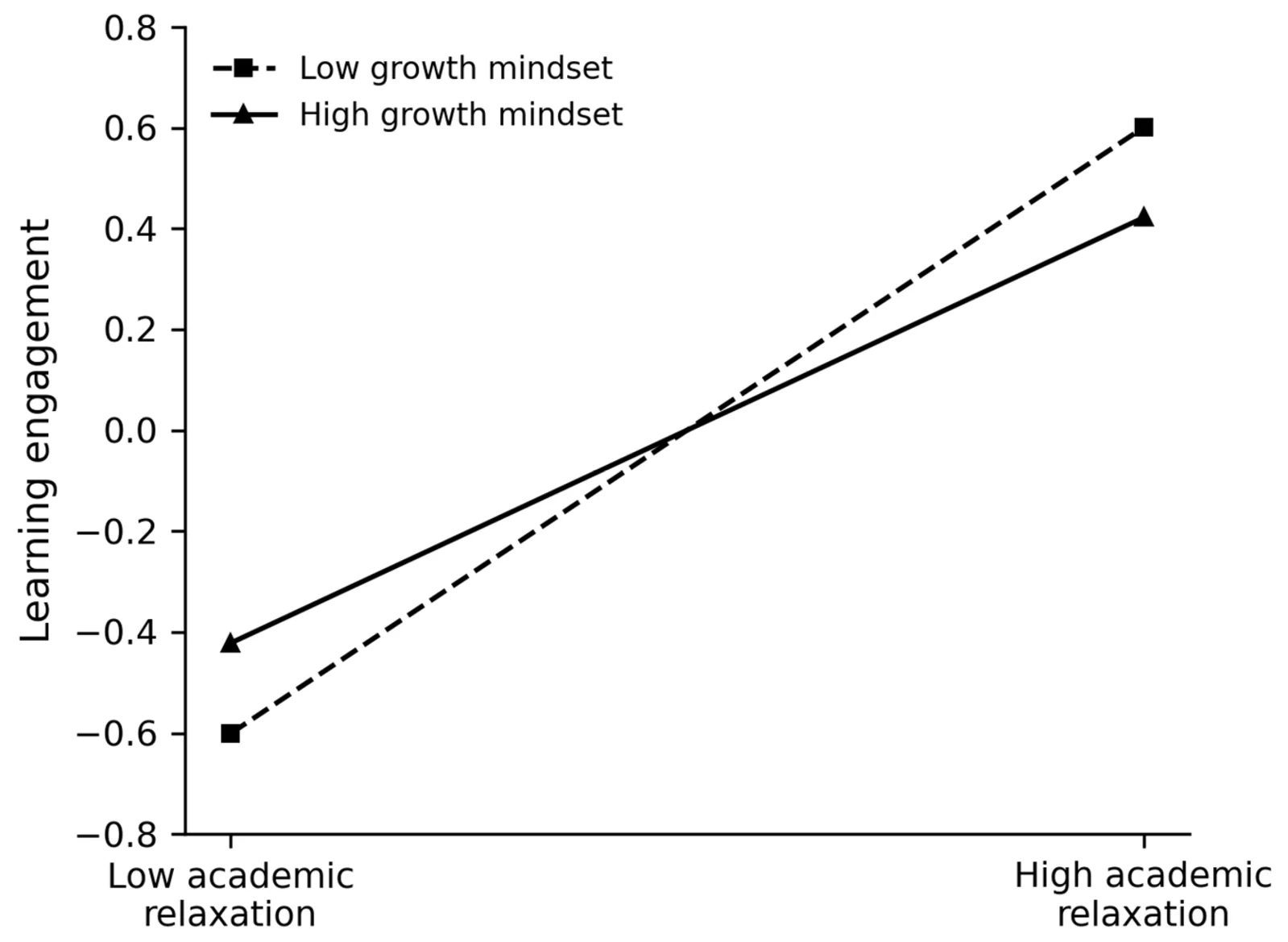
Moderating Role of Growth Mindset in the Association Between Academic Relaxation and Mathematics Learning Engagement. Note. All continuous variables were standardized. Low and high growth mindset represent −1 SD and +1 SD from the mean, respectively.

The interaction between academic relaxation and growth mindset was significant and negative, *B* = −0.086, HC3 *SE* = 0.028, *z* = −3.099, *p* = 0.002, 95% CI [−0.140, −0.032]. Academic relaxation was positively associated with engagement at both low growth mindset, *B* = 0.574, HC3 *SE* = 0.041, *z* = 13.992, *p* < 0.001, 95% CI [0.494, 0.655], and high growth mindset, *B* = 0.402, HC3 *SE* = 0.037, *z* = 10.890, *p* < 0.001, 95% CI [0.330, 0.475]. Thus, academic relaxation remained positively associated with engagement at both levels, but the association was stronger among students with lower growth mindset. Because the direction of the interaction was opposite to that predicted, Hypothesis 4c was not supported (Figure 5).

### 4.8. Screening Sensitivity Analysis

The principal effects were generally robust to alternative response-pattern screening criteria and to post hoc exclusions of very short online completion times. The positive association between perceived teacher autonomy support and mathematics learning engagement and all three indirect effects remained statistically significant across the five screening specifications. The perceived teacher autonomy support × growth mindset interaction remained positive and significant, whereas the academic relaxation × growth mindset interaction remained negative and significant. The beliefs about errors × growth mindset interaction was not significant in the primary analysis and became significant only when online completion times below 90 s were excluded; this interaction was therefore treated as screening-sensitive rather than robust.

## 5. Discussion

### 5.1. Perceived Teacher Autonomy Support Is Positively Associated with Mathematics Learning Engagement

After gender and school stage were controlled, perceived teacher autonomy support was positively associated with mathematics learning engagement. This finding is consistent with SDT, which proposes that students are more likely to engage actively when teachers acknowledge their perspectives, provide meaningful choices, explain the value of learning tasks, and minimize controlling demands (Ryan & Deci, 2000, 2017). It is also consistent with previous intervention, longitudinal, and correlational studies reporting positive links between teacher autonomy support and student engagement (Chen et al., 2015; Jiang et al., 2023; Reeve et al., 2004; Yu et al., 2016). The present cross-sectional result should, however, be interpreted as an association rather than evidence that perceived autonomy support causally increases engagement.

These findings indicate that teacher autonomy support is an important classroom condition for students’ learning engagement. Its role does not lie in weakening teacher guidance, but in enhancing students’ sense of agency in learning through supportive interaction. When students feel understood, respected, and given appropriate choices in the classroom, they are more likely to perceive learning tasks as meaningful, participatory, and controllable, thereby showing higher levels of learning engagement.

### 5.2. Beliefs About Errors and Academic Relaxation Show Serial Indirect Associations 

This study found significant specific and serial indirect associations involving beliefs about errors and academic relaxation. The findings suggest that the association between perceived teacher autonomy support and mathematics learning engagement may involve both students’ interpretations of errors and their emotional experiences during mathematics learning. Because the data were cross-sectional, these indirect associations should not be interpreted as evidence of a temporal or causal mechanism. In mathematics learning, errors commonly arise during problem solving, proof construction, and conceptual understanding. An autonomy-supportive classroom environment may be associated with students’ interpreting errors as diagnosable and correctable information. Such interpretations may, in turn, be associated with lower perceived threat, greater academic relaxation, and sustained engagement. This explanation is theoretically plausible but requires longitudinal or experimental verification.

Existing research has shown that positive beliefs about errors can predict students’ adaptive emotional, motivational, and behavioral responses after errors, and that students’ emotional experiences after failure in mathematics are closely related to how they understand errors and failure (Tulis & Ainley, 2011; Tulis et al., 2018). Research on academic emotions further indicates that emotions affect students’ cognitive resources, learning strategies, and self-regulation processes. Control-value theory also emphasizes that students’ perceived control over, and value attached to, learning activities are important antecedents of academic emotions and subsequent learning behavior (Pekrun, 2006; Pekrun et al., 2002).

The contribution of this study lies in examining beliefs about errors and academic relaxation within the same serial model. The findings suggest that the association between perceived teacher autonomy support and mathematics learning engagement involves not only students’ willingness to participate but also how they interpret errors and experience emotional threat during mathematics learning.

### 5.3. Growth Mindset Moderates the Association Between Perceived Teacher Autonomy Support and Mathematics Learning Engagement

This study found that growth mindset positively moderated the association between teacher autonomy support and mathematics learning engagement. Compared with students with lower levels of growth mindset, the positive association between perceived teacher autonomy support and mathematics learning engagement was stronger among students with higher levels of growth mindset. This pattern suggests that students with a stronger growth mindset may be better able to interpret autonomy-supportive teaching as an opportunity for developing their mathematical ability. Because the study was cross-sectional, this interpretation should not be treated as evidence of a causal resource-conversion process.

This finding is consistent with growth mindset theory. Dweck and Leggett (1988) argued that beliefs about the malleability of ability shape individuals’ goal orientations and responses to difficulty. Students with a stronger growth mindset may interpret teachers’ choices, supportive feedback, and non-controlling guidance as opportunities for developing their ability and may therefore make greater use of autonomy-supportive classroom experiences. This interpretation is also consistent with research linking growth mindset with effort beliefs, adaptive attribution, and self-regulatory processes (Blackwell et al., 2007; Burnette et al., 2013). Nevertheless, because the present study was cross-sectional, the result should not be interpreted as evidence of a causal resource-conversion process.

### 5.4. Growth Mindset Did Not Robustly Moderate the Beliefs-About-Errors Pathway

Beliefs about errors were positively associated with mathematics learning engagement, but growth mindset did not significantly moderate this association in the primary full-sample HC3 analysis. Hypothesis 4b was therefore not supported. The conditional association was positive at both low and high levels of growth mindset, suggesting that regarding errors as useful learning information may be relevant to engagement across different levels of growth mindset. Across the alternative screening specifications, the interaction remained nonsignificant in most analyses and reached statistical significance only when online completion times below 90 s were excluded. The moderation result should therefore be regarded as screening-sensitive rather than robust and requires replication in independent samples.

### 5.5. Growth Mindset Negatively Moderates the Relationship Between Academic Relaxation and Mathematics Learning Engagement

This study found that growth mindset negatively moderated the relationship between academic relaxation and mathematics learning engagement. Specifically, academic relaxation remained positively associated with learning engagement, but this association was stronger among students with lower levels of growth mindset and relatively weaker among students with higher levels of growth mindset. This result is inconsistent with the original hypothesis, indicating that growth mindset does not play a homogeneous strengthening role across all pathways.

One possible interpretation is that students with lower levels of growth mindset may rely more strongly on a calm and low-threat emotional state when sustaining engagement. Students with higher growth mindset may draw on additional motivational or self-regulatory resources when facing difficulty. However, this interpretation was developed after observing the unexpected interaction pattern and should therefore be regarded as speculative rather than as a mechanism established by the present study. Longitudinal and experimental studies are needed to examine this possibility.

Growth mindset did not reduce the positive value of academic relaxation itself; rather, it altered the strength of the association between academic relaxation and engagement. The results therefore indicate a path-specific pattern: growth mindset strengthened the association between perceived teacher autonomy support and engagement, did not robustly moderate the beliefs-about-errors pathway, and weakened the positive association between academic relaxation and engagement.

### 5.6. Limitations and Future Research

This study has several limitations. First, the cross-sectional survey design does not permit causal or temporal conclusions. Longitudinal, experimental, and classroom-process studies are needed to examine the ordering of perceived teacher autonomy support, beliefs about errors, academic relaxation, and engagement. Second, all core constructs were assessed through student self-reports collected at one time point. Although the five-factor model fitted the data substantially better than the single-factor model, the analyses did not eliminate the possibility of common method variance. Future studies should combine student reports with classroom observations, teacher reports, and behavioral indicators.

Third, the study focused on academic relaxation as a low-arousal positive emotion. Students may simultaneously experience anxiety, shame, boredom, hope, and other emotions in error-related situations. Future research should examine multiple emotions and possible nonlinear associations between relaxation and engagement.

Fourth, students may have been exposed to shared classroom and school contexts. However, the original sampling and data-management procedures did not use a school- or class-level sampling frame and did not collect or retain anonymized school or class identifiers. Consequently, the number of participating schools and classes could not be reconstructed, and intraclass correlations, cluster-robust standard errors, and multilevel models could not be estimated. Although gender and school stage were controlled and HC3 standard errors were used to address heteroscedasticity, these procedures do not account for possible classroom- or school-level dependency. The precision of the reported standard errors should therefore be interpreted with caution.

Fifth, although the original questionnaires were retrieved and the retained and straight-line-excluded respondents were compared, the groups differed in gender, school stage, survey mode, and online completion time. The demographic differences were small in magnitude, but the excluded respondents were more likely to be male, to have completed the survey online, and to have substantially shorter completion times. These differences suggest that the analytic sample may not fully represent all returned questionnaires and that response quality was associated with the survey-administration context.

## 6. Conclusions

The present study showed that perceived teacher autonomy support was positively associated with mathematics learning engagement, both directly and indirectly through beliefs about errors and academic relaxation. The results supported a serial indirect pathway in which perceived autonomy support was associated with more adaptive interpretations of errors, which were in turn associated with a calmer learning state and greater engagement. Growth mindset showed path-specific moderation: it strengthened the association between perceived teacher autonomy support and engagement but did not robustly moderate the beliefs-about-errors pathway. In addition, the positive association between academic relaxation and engagement was stronger among students with lower growth mindset. These findings highlight the importance of autonomy-supportive teaching, constructive interpretations of errors, and emotionally low-threat mathematics-learning environments.

## Figures and Tables

**Table 1:** Measurement Model Comparisons.

Model	*χ* ^2^	*df*	CFI	TLI	RMSEA	90% CI	SRMR
Five-factor model	1288.713	179	0.988	0.986	0.061	[0.058, 0.064]	0.028
Four-factor: relaxation + engagement	4088.096	183	0.957	0.950	0.113	[0.110, 0.116]	0.048
Four-factor: errors + engagement	5757.808	183	0.938	0.929	0.135	[0.132, 0.138]	0.060
Single-factor model	15,915.004	189	0.826	0.806	0.223	[0.220, 0.226]	0.127

Note. CFI = comparative fit index; TLI = Tucker–Lewis index; RMSEA = root mean square error of approximation; CI = confidence interval; SRMR = standardized root mean square residual.

**Table 2:** Reliability and Convergent Validity of the Study Measures.

Construct	Items	α	ω/CR	AVE
Perceived teacher autonomy support	3	0.716	0.827	0.625
Beliefs about errors	5	0.933	0.952	0.798
Academic relaxation	5	0.921	0.941	0.760
Mathematics learning engagement	3	0.934	0.954	0.873
Growth mindset	5	0.865	0.911	0.674

Note. α = Cronbach’s alpha; ω = McDonald’s omega; CR = composite reliability; AVE = average variance extracted.

**Table 3:** Descriptive Statistics and Correlations Among the Study Variables.

Variable	*M*	*SD*	1	2	3	4	5
1. Perceived teacher autonomy support	3.855	0.842	1				
2. Beliefs about errors	3.782	0.869	0.482 **	1			
3. Academic relaxation	3.338	0.950	0.344 **	0.562 **	1		
4. Mathematics learning engagement	3.513	1.005	0.480 **	0.669 **	0.751 **	1	
5. Growth mindset	4.029	0.821	0.300 **	0.493 **	0.540 **	0.513 **	1

Note. *N* = 1679. *M* = mean; *SD* = standard deviation. ** *p* < 0.01.

**Table 4:** HC3 Regression Results for the Serial Mediation Model.

Outcome	Predictor	*B*	HC3 *SE*	*z*	*p*	95% CI	*R* ^2^
Beliefs about errors	TAS	0.491	0.028	17.567	<0.001	[0.436, 0.546]	0.272
Academic relaxation	TAS	0.129	0.031	4.190	<0.001	[0.069, 0.189]	0.381
	Beliefs about errors	0.505	0.031	16.388	<0.001	[0.445, 0.565]	
Engagement	TAS	0.196	0.026	7.463	<0.001	[0.144, 0.247]	0.677
	Beliefs about errors	0.333	0.034	9.915	<0.001	[0.267, 0.398]	
	Academic relaxation	0.538	0.030	17.976	<0.001	[0.479, 0.597]	

Note. Gender and school stage were included as covariates but are omitted from the table for concision.

**Table 5:** Direct and Indirect Associations in the Serial Mediation Model.

Effect	Estimate	Boot SE	95% Bootstrap CI
Total effect	0.562	—	[0.509, 0.615]
Direct effect	0.196	—	[0.144, 0.247]
Total indirect effect	0.366	0.025	[0.316, 0.415]
TAS → Errors → Engagement	0.163	0.020	[0.125, 0.204]
TAS → Relaxation → Engagement	0.069	0.018	[0.036, 0.106]
TAS → Errors → Relaxation → Engagement	0.134	0.013	[0.109, 0.159]

Note. Path 1 = teacher autonomy support → beliefs about errors → learning engagement; Path 2 = teacher autonomy support → academic relaxation → learning engagement; Path 3 = teacher autonomy support → beliefs about errors → academic relaxation → learning engagement.

**Table 6:** HC3 Regression Results for the Moderated Serial Mediation Model.

Predictor	*B*	HC3 *SE*	*z*	*p*	95% CI
TAS	0.174	0.024	7.405	<0.001	[0.128, 0.220]
Beliefs about errors	0.280	0.029	9.503	<0.001	[0.222, 0.338]
Academic relaxation	0.488	0.028	17.732	<0.001	[0.434, 0.542]
Growth mindset	0.031	0.020	1.546	0.122	[−0.008, 0.070]
TAS × Growth mindset	0.040	0.018	2.193	0.028	[0.004, 0.075]
Errors × Growth mindset	0.044	0.027	1.650	0.099	[−0.008, 0.097]
Relaxation × Growth mindset	−0.086	0.028	−3.099	0.002	[−0.140, −0.032]
Gender	−0.039	0.028	−1.363	0.173	[−0.094, 0.017]
Stage 2	−0.038	0.031	−1.224	0.221	[−0.100, 0.023]
Stage 3	−0.155	0.037	−4.197	<0.001	[−0.228, −0.083]

**Table 7:** Conditional Indirect Associations and Indices of Moderated Mediation.

**Panel A. Conditional indirect associations**				
**Growth Mindset**	**Indirect Path**	**Effect**	**Boot SE**	**95% CI**
−1 SD	TAS → Errors → Engagement	0.112	0.020	[0.075, 0.154]
Mean	TAS → Errors → Engagement	0.133	0.017	[0.101, 0.169]
+1 SD	TAS → Errors → Engagement	0.154	0.022	[0.111, 0.199]
−1 SD	TAS → Relaxation → Engagement	0.066	0.017	[0.033, 0.101]
Mean	TAS → Relaxation → Engagement	0.056	0.014	[0.029, 0.085]
+1 SD	TAS → Relaxation → Engagement	0.046	0.012	[0.023, 0.071]
−1 SD	TAS → Errors → Relaxation → Engagement	0.126	0.013	[0.102, 0.154]
Mean	TAS → Errors → Relaxation → Engagement	0.107	0.011	[0.088, 0.129]
+1 SD	TAS → Errors → Relaxation → Engagement	0.089	0.011	[0.068, 0.111]
**Panel B. Indices of moderated mediation**				
**Path**		**Index**	**Boot SE**	**95% CI**
TAS → Errors → Engagement		0.021	0.013	[−0.005, 0.045]
TAS → Relaxation → Engagement		−0.010	0.004	[−0.019, −0.003]
TAS → Errors → Relaxation → Engagement		−0.019	0.006	[−0.031, −0.007]

## Data Availability

The raw data supporting the conclusions of this article will be made available by the corresponding author upon reasonable request, without undue reservation.

## Appendix A

**Table A1:** Comparison of Retained and Straight-Line-Excluded Respondents.

Characteristic	Retained Sample, *n* = 1679	Straight-Line-Excluded Sample, *n* = 717	Test Statistic	*p*	Effect Size
Gender			Pearson χ^2^(1) = 23.55	<0.001	Cramér’s *V* = 0.099
Male	769 (45.8%)	406 (56.6%)			
Female	910 (54.2%)	311 (43.4%)			
School stage			Pearson χ^2^(2) = 18.61	<0.001	Cramér’s *V* = 0.088
Primary/preparatory	692 (41.2%)	343 (47.8%)			
Junior secondary	515 (30.7%)	159 (22.2%)			
Senior secondary/vocational	472 (28.1%)	215 (30.0%)			
Survey mode			Pearson χ^2^(1) = 41.59	<0.001	Cramér’s *V* = 0.132
Online administration	1570 (93.5%)	714 (99.6%)			
Paper-and-pencil administration	109 (6.5%)	3 (0.4%)			
Online completion time, median	165 s	81 s	Mann–Whitney *U* = 884,506.5	<0.001	Rank-biserial *r* = 0.578

Note. Values are presented as *n* (%) unless otherwise indicated. Percentages are column percentages. Pearson chi-square tests were used to compare gender, school stage, and survey mode. The completion-time analysis included online respondents only (retained sample, *n* = 1570; straight-line-excluded sample, *n* = 714). *V* = Cramér’s V; *r* = rank-biserial correlation. All tests were two-sided.
